# Ocular surface immune transcriptome and tear cytokines in corneal infection patients

**DOI:** 10.3389/fcimb.2024.1346821

**Published:** 2024-04-17

**Authors:** Heba Alenezi, Grant Parnell, Stephen Schibeci, Jerome Ozkan, Mark Willcox, Andrew J. R. White, Nicole Carnt

**Affiliations:** ^1^ Department of Medical Laboratory Sciences, College of Applied Medical Sciences in Al-Kharj, Prince Sattam Bin Abdulaziz University, Al-Kharj, Saudi Arabia; ^2^ School of Optometry and Vision Science, The University of New South Wales, Sydney, NSW, Australia; ^3^ Centre for Vision Research, Westmead Institute for Medical Research, The University of Sydney, Sydney, NSW, Australia; ^4^ Centre for Immunology and Allergy Research, Westmead Institute for Medical Research, The University of Sydney, Sydney, NSW, Australia; ^5^ School of Medical Sciences, Faculty of Medicine and Health, The University of Sydney, Sydney, NSW, Australia; ^6^ Institute of Ophthalmology, University College London, London, United Kingdom

**Keywords:** corneal infection, conjunctiva, ocular surface, keratitis, bacteria, transcriptome, gene expression

## Abstract

**Background:**

Microbial keratitis is one of the leading causes of blindness globally. An overactive immune response during an infection can exacerbate damage, causing corneal opacities and vision loss. This study aimed to identify the differentially expressed genes between corneal infection patients and healthy volunteers within the cornea and conjunctiva and elucidate the contributing pathways to these conditions’ pathogenesis. Moreover, it compared the corneal and conjunctival transcriptomes in corneal-infected patients to cytokine levels in tears.

**Methods:**

Corneal and conjunctival swabs were collected from seven corneal infection patients and three healthy controls under topical anesthesia. RNA from seven corneal infection patients and three healthy volunteers were analyzed by RNA sequencing (RNA-Seq). Tear proteins were extracted from Schirmer strips via acetone precipitation from 38 cases of corneal infection and 14 healthy controls. The cytokines and chemokines IL-1β, IL-6, CXCL8 (IL-8), CX3CL1, IL-10, IL-12 (p70), IL-17A, and IL-23 were measured using an antibody bead assay.

**Results:**

A total of 512 genes were found to be differentially expressed in infected corneas compared to healthy corneas, with 508 being upregulated and four downregulated (fold-change (FC) <−2 or > 2 and adjusted p <0.01). For the conjunctiva, 477 were upregulated, and 3 were downregulated (FC <−3 or ≥ 3 and adjusted p <0.01). There was a significant overlap in cornea and conjunctiva gene expression in patients with corneal infections. The genes were predominantly associated with immune response, regulation of angiogenesis, and apoptotic signaling pathways. The most highly upregulated gene was *CXCL8* (which codes for IL-8 protein). In patients with corneal infections, the concentration of IL-8 protein in tears was relatively higher in patients compared to healthy controls but did not show statistical significance.

**Conclusions:**

During corneal infection, many genes were upregulated, with most of them being associated with immune response, regulation of angiogenesis, and apoptotic signaling. The findings may facilitate the development of treatments for corneal infections that can dampen specific aspects of the immune response to reduce scarring and preserve sight.

## 1 Introduction

Microbial keratitis is a serious infection of the cornea that classically presents with an epithelial defect with underlying infiltration by white blood cells (mostly neutrophils). During healing, microbial keratitis can give rise to corneal opacities due to scarring, which can result in blindness. Keratitis is one of the leading causes of blindness globally (Pascolini and Mariotti, 2012; Upadhyay et al., 2015; Ung et al., 2019). Pathogens can invade the cornea and cause infection when corneal defense mechanisms are decreased, such as after epithelium damage (Bourcier et al., 2003; Santacruz et al., 2015). Common identified risk factors for microbial keratitis are contact lens wear, ocular trauma, ocular surface diseases, previous ocular surgery, diabetes mellitus and immunosuppression (Bourcier et al., 2003; Keay et al., 2006; Al-Mujaini et al., 2009; Zimmerman et al., 2016; Tena et al., 2019). A wide spectrum of microorganisms can cause microbial keratitis, but bacteria represent the most common cause (Musa et al., 2010; Das et al., 2019; Green et al., 2019; Tena et al., 2019; Chen et al., 2020). Most prevalent among the bacteria are the Gram‐positive *Streptococcus pneumoniae, Staphylococcus aureus* and coagulase-negative *staphylococci*, and the Gram‐negative bacterium *Pseudomonas aeruginosa* (Orlans et al., 2011; Hoddenbach et al., 2014; O’Neill et al., 2014; Das et al., 2019; Green et al., 2019; Cabrera-Aguas et al., 2020).

During microbial keratitis, excessive activation of the host’s immune response contributes to tissue destruction, corneal opacities and vision loss (Willcox, 2007). Bacteria such as *Pseudomonas aeruginosa* produce virulence factors that can activate toll-like receptors (TLRs) on corneal epithelial cells (Willcox, 2007). TLRs recognize bacterial components such as lipopolysaccharide (LPS) or flagella from *P. aeruginosa* (Takeuchi et al., 1999; Barton and Medzhitov, 2003). Several TLRs (TLR2, TLR3, TLR4, TLR5, TLR8, and TLR9) are expressed by corneal epithelial cells (Zhang et al., 2003; Johnson et al., 2005; Huang et al., 2006; Mohammed et al., 2011; Chidambaram et al., 2017; Ueta et al., 2019). TLR2 is the main receptor for Gram-positive bacteria, while TLR4 regulates the LPS response (Hoshino et al., 1999; Takeuchi et al., 1999). The activation of TLRs stimulates epithelial cells to produce chemokines and cytokines (Willcox, 2007) such as interleukin-8 (IL-8), interleukin-1β (IL-1β) and interleukin-6 (IL-6) (Xue et al., 2000). These cytokines and chemokines amplify and control the response to infection by recruiting neutrophils and other white blood cells to the site of infection to eradicate the bacteria (Hazlett, 2005; Willcox, 2007). However, the persistent presence of neutrophils, and their defense systems such as enzymes and the reactive oxygen radicals can cause significant corneal damage and scarring (Chusid and Davis, 1979; Thakur et al., 2001).

Gene expression, particularly differential expression data for normal and disease tissues, may provide information that could help understand the disease’s pathogenic mechanisms. During microbial corneal infection, the host response follows particular biological pathways as the disease advances (Hazlett, 2004; Karthikeyan et al., 2013; Santacruz et al., 2015; Shrestha et al., 2021). However, there is a lack of information on the exact molecular mechanisms involved in these pathways in humans. To understand the immunopathogenesis of microbial corneal infection, this study analyzed the transcriptomes of the cornea and conjunctiva using RNA-seq of individuals with microbial infections compared to healthy controls.

## 2 Materials and methods

### 2.2 Ethics statement

The study was approved by the Western Sydney Local Health District Human Research Ethics Committee (LNR/16/WMEAD/401), and the research followed the tenets of the Declaration of Helsinki. All participants signed informed consent prior to study commencement.

### 2.3 Participant enrolment and sample collection

Corneal infection patients undergoing a corneal scrape were recruited at the time of initial presentation at Westmead Hospital, Sydney, Australia. Healthy volunteers were recruited from the School of Optometry and Vision Science, University of New South Wales, Sydney, Australia. The cornea and conjunctiva swabs (Puritan Medical Products, ME, USA) were performed under topical anesthesia (oxybuprocaine hydrochloride 0.4% 4mg/mL minim eye drops, Bausch & Lomb, Australia). The affected eye was used for patients with corneal infection, while for healthy volunteers, the sampled eye was randomized. The individual swabs were placed in separate sterile microcentrifuge tubes containing 400 μl RNAlater^®^ (R0901, Sigma-Aldrich) and stored at – 80°C until RNA extraction. Swabs and then tears were collected for patients following the standard diagnostic corneal scrape. Tears were collected from the same eye after swabbing, with Schirmer strips (HUB Pharmaceuticals, MI, USA) and stored at – 80°C until analysis. During the diagnostic corneal scrape, samples were collected from the corneal infection by a sterile 23-gauge needle or D25 scalpel and were plated in the clinic onto various media for culture in the diagnostic laboratory.

The present study involved the acquisition and analysis of RNA sequences from swab samples of seven patients with corneal infection and three healthy controls. In addition to the RNA cohort, tear samples were collected and analyzed from 31 further cases of corneal infection and 11 healthy controls, as detailed in **Table 1**.

**Table 1:** Demographics of the study participants.

	RNA sequences			Tear cytokine analysis		
	Cases (n=7)	Controls (n=3)	P value	Cases (n=38)	Controls (n=14)	P-value
Female, n (%)	4 (57%)	2 (67%)	0.8	16 (42%)	8 (57%)	0.3
Age, mean ± SD	58 ± 30	37 ± 4	0.3	48 ± 18	42 ± 13	0.3

SD, Standard deviation.

### 2.4 RNA isolation

Total RNA extraction of the corneal and conjunctival swabs was performed using NucleoSpin, RNA XS (Macherey-Nagel, Düren, Germany), following the manufacturer’s instructions. The RNA quality was checked in representative samples using the Agilent 2100 Bioanalyzer (Agilent Technologies, USA). The RNA purity and integrity of the samples were assessed according to the RNA integrity number (RIN), which can range from one (completely degraded) to 10 (fully intact) (Mueller et al., 2004; Schroeder et al., 2006). The RIN obtained was higher than seven in all samples measured and deemed suitable for RNA sequencing.

### 2.5 Library preparation and RNA-Seq

Sequencing libraries were prepared from 2 ng total RNA from each sample using the SMARTer Stranded Total RNA-Seq Kit v2 − Pico Input Mammalian (Takara Bio USA, Mountain View, CA, USA), according to the manufacturer’s protocol. The samples were processed in two batches; for the first batch, single-end (100 bp) library sequencing was performed on the Illumina HiSeq2500 Sequencing System (Illumina, San Diego, CA, USA), with three corneal infection and three control samples. Paired-end (50 bp) library sequencing was performed on the Illumina NovaSeq 6000 Sequencing System (Illumina, San Diego, CA, USA) for batch two, with four corneal infection samples.

### 2.6 Read mapping and differential expression analysis

The raw sequencing data was processed using Trimmomatic 0.39 (Bolger et al., 2014) to filter out low-quality reads and any data containing adapter/primer sequences. The filtered reads were mapped to the Homo sapiens genome (GRCh38.p13) using HISAT2 (v 2.2.1) (Kim et al., 2019). In the first batch, the number of mapped reads per sample ranged from 0.5 million to 29.8 million, while in the second batch, the range was 2.3 million to 77.3 million. To reduce uninformative variation and normalize sequencing depth between the samples, the median of ratios function provided by DESeq2 has been utilized (Anders and Huber, 2010). The PCA function of the (DESeq. 2 Bioconductor package) was used to perform principal component analysis. The expression level of genes for all the corneal infection samples and the controls was used to determine the principal components analysis (PCA) division of the corneal infection cases group and the healthy control group. Differential gene expression was calculated using the Bioconductor package, DESeq. 2 (v1.30.1) (Love et al., 2014). The differential gene expression thresholds were set with reference to the number of genes. Differential gene expression was calculated for the corneal samples based on the threshold criterion of log fold change > 1 (fold change (FC) <−2 or > 2) and adjusted P < 0.01. The differential gene expression for the conjunctiva samples was calculated based on the threshold criterion of log fold change (FC) > 1.58 (FC <−3 or ≥ 3) and adjusted P < 0.01. Due to the larger number of differentially expressed genes in the conjunctiva, a more stringent threshold for fold change was applied to obtain a manageable number of genes for downstream analysis. Gene IDs for the differentially expressed genes were linked to gene-specific information using the National Center for Biotechnology Information (NCBI) database (https://www.ncbi.nlm.nih.gov/gene).

### 2.7 Functional analysis

Functional annotations for the differentially expressed genes (DEGs) were performed using the Bioconductor package, clusterProfiler (v3.18.1) (Yu et al., 2012; Wu et al., 2021). The enrichGO and the enrichKEGG function from the clusterProfiler Bioconductor package were used to perform the enrichment analysis of Gene Ontology (GO) functions and the Kyoto Encyclopedia of Genes and Genomes (KEGG) pathway, respectively. The cut-off criteria of q value (corrected p-value) < 0.05 were used to select the significantly enriched GO terms and genes’ significantly enriched KEGG pathway.

### 2.8 Tear cytokine analysis

The cytokines and chemokines IL-1β, IL-6, IL-8, CX3CL1, IL-10, IL-12 (p70), IL-17A and IL-23 were measured in the tear samples of 38 corneal infection cases and 14 healthy controls. Tear proteins were extracted from Schirmer strips using acetone precipitation for maximum yield (Powell et al., 2010) The measurement was carried out in duplicate using Millipore’s MILLIPLEX^®^ MAP Human High Sensitivity T Cell Magnetic Bead Panel kit (Millipore Corporation, Billerica, MA, USA) and a Luminex 100/200 system (Luminex Corporation, Austin, TX). The kit reagent was used to dilute the samples considering the sample volume and normalized in preparation for analysis. Standard curves for the Luminex assay were produced utilizing known duplicate dilutions. Samples that contained concentrations lower than the detection limit were designated as not detectable, while those exceeding the lower detection limit were adjusted according to the dilution factor. The data were analyzed using GraphPad Prism 10 software (GraphPad Software, La Jolla, CA). As a crucial step in data analysis, the Robust Regression and Outlier Removal (ROUT) methodology has effectively identified outliers with a Q value of 0.1% (Motulsky and Brown, 2006). It should be noted that Q denotes the maximum acceptable false discovery rate. Unpaired samples were compared using the Mann-Whitney test, a non-parametric method. The results have been adjusted for the multiple comparisons utilizing the Bonferroni method. A two-tailed p-value of less than 0.05 determined significance and the results were reported as median & interquartile ranges (IQR).

## 3 Results

### 3.1 Overview of RNA-Seq samples

Of seven corneal infection samples, one case was identified as polymicrobial with the presence of *Staphylococcus aureus, coagulase-negative Staphylococcus*, and Varicella-Zoster virus (VZV) cultured in the diagnostic microbiology laboratory of the hospital from the corneal scrapes. One infection was caused by *Streptococcus pneumonia*. Cultured scrapes from three cases were culture negative. Data on the microbes could not be retrieved for the remaining two cases.

The RNA-Seq data for all the samples were subjected to principal components analysis (**Supplementary Figure 1**) and showed cornea and conjunctiva infection samples clustered together and the control samples clustered together. There was no discernible differentiation between the samples obtained from the cornea and those collected from the conjunctiva. This consistency was found to be the case across different batches. In addition, differential expression analysis identified several differentially expressed genes (DEGs) in the cornea and conjunctiva infection samples compared to healthy volunteers.

### 3.2 Common differentially expressed genes of the cornea and conjunctiva during corneal infection

**Figure 1:**
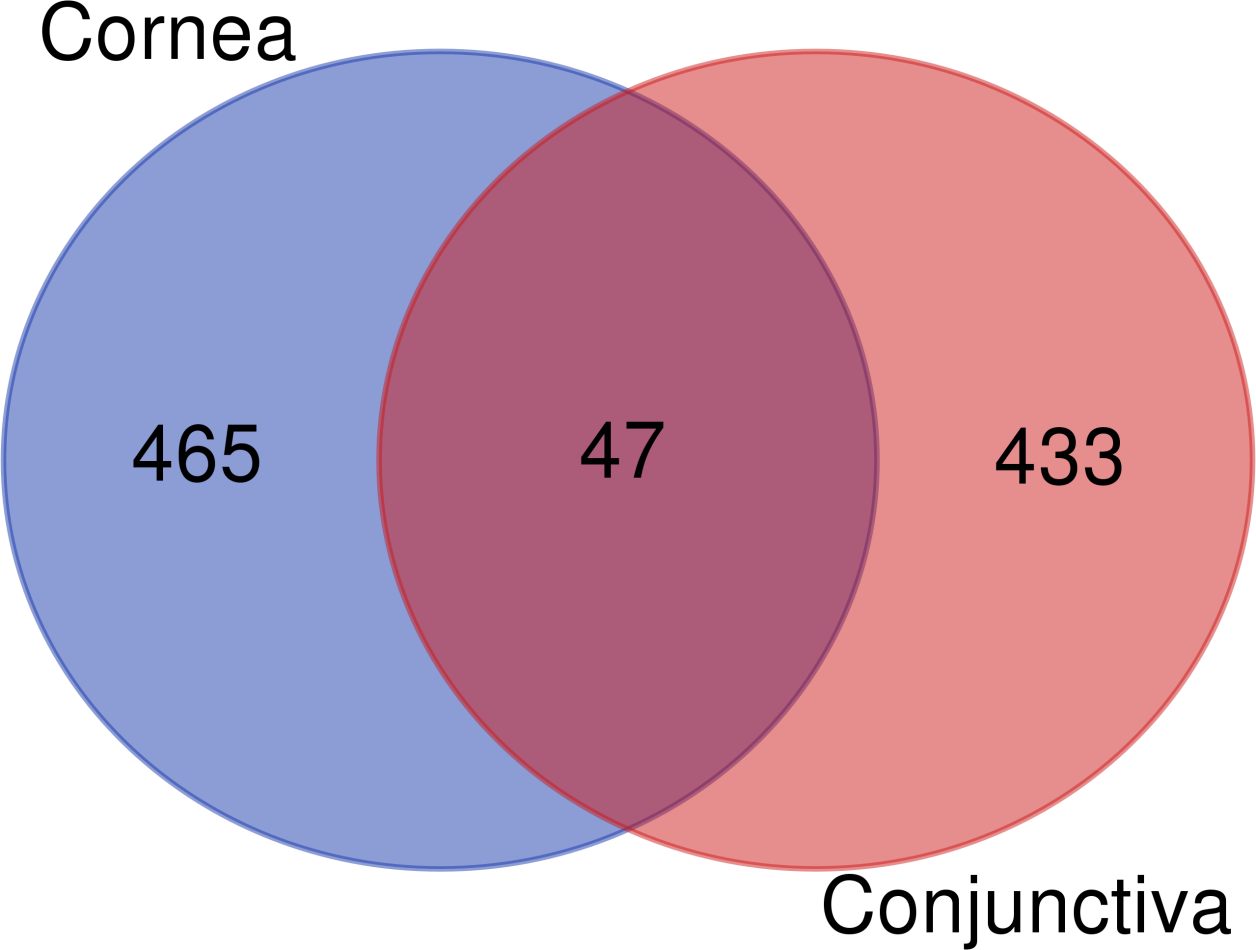
Venn diagram of differentially expressed genes in the cornea and conjunctiva.

In the cornea and conjunctiva of patients with corneal infection compared to controls, 945 differentially expressed genes (DEGs) were identified at a significance level of adjusted P < 0.01. Among these DEGs, 465 were differentially expressed in the cornea only, 433 were differentially expressed in the conjunctiva only, and 47 were differentially expressed in both tissues (**Figure 1**) (**Supplementary Table S1**).

**Figure 2:**
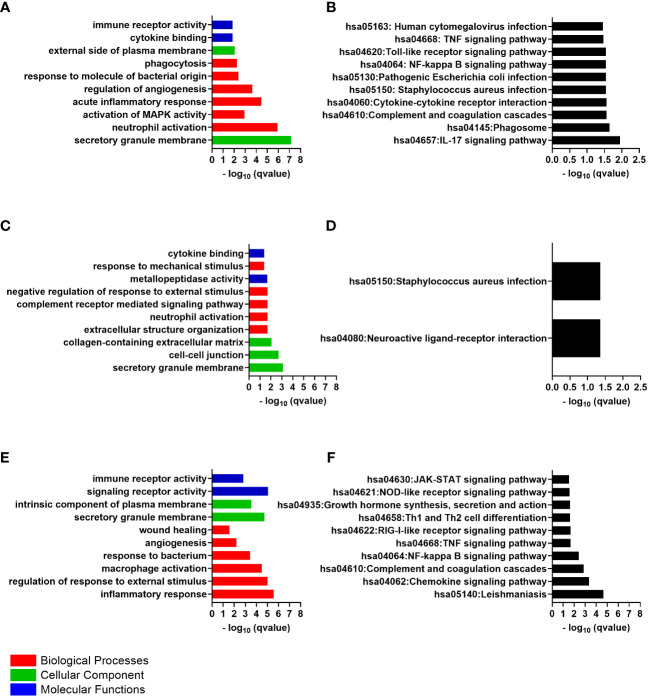
The top 10 gene ontology (GO) terms and Kyoto Encyclopedia of Genes and Genomes (KEGG) pathways of differentially expressed genes, ranked by their -log p values. **(A)** Common GO Enrichment analysis; **(B)** Common KEGG pathway enrichment analysis; **(C)** Cornea GO Enrichment analysis; **(D)** Cornea KEGG pathway enrichment analysis; **(E)** Conjunctiva GO Enrichment analysis; **(F)** Conjunctiva KEGG pathway enrichment analysis.

The 47 differentially expressed genes common to the cornea and conjunctiva, which were all upregulated genes, were analyzed to investigate their biological function. The enrichGO function from the clusterProfiler Bioconductor package was used to identify significantly enriched Gene Ontology (GO)terms. The cut-off criteria used for this analysis was a q-value of < 0.05. Gene Ontology is divided into three categories: Biological Process (BP), Cellular Component (CC), and Molecular Function (MF). The most significantly enriched GO terms in the Biological Process category were those for neutrophil activation, leukocyte migration, acute inflammatory response, response to molecule of bacterial origin, phagocytosis, humoral immune response, and cytokine secretion (**Figure 2A**).

The particular genes that were found to be enriched included C-X-C motif chemokine ligand 8 (*CXCL8*) and its receptor C-X-C motif chemokine receptor 2 (*CXCR2*), interleukin 1 beta (*IL1B*), triggering receptor expressed on myeloid cells 1 (*TREM1* also called *CD354*), complement C5a receptor 1 (*C5AR1* also called *CD88*), colony-stimulating factor 3 receptor (*CSF3R* also called *CD114*), solute carrier family 11 member 1 (*SLC11A1*), adhesion G protein-coupled receptor E3 (*ADGRE3*), Fos proto-oncogene, AP-1 transcription factor subunit (*FOS* also called or *p55*), serglycin (*SRGN*) and NADPH oxidase complex subunit neutrophil cytosolic factor 2 (*NCF2*). In addition, cell adhesion molecules such as integrin subunit alpha X (*ITGAX* also called *CD11C*) and selectin L (*SELL* also called *CD62L*) were also enriched in these immune response terms.

Numerous terms were also significantly enriched in the “biological process”, including the regulation of angiogenesis and apoptotic signaling pathway. Within these terms, genes such as thrombospondin 1 (*THBS1*), prostaglandin-endoperoxide synthase 2 (*PTGS2*), plasminogen activator, urokinase receptor (*PLAUR* also called *CD87*), and G0/G1 switch 2 (*G0S2*) were enriched. Other enriched terms in the biological process included activation of MAPK activity, activation of protein kinase activity, positive regulation of protein serine/threonine kinase activity, response to cAMP, and positive regulation of reactive oxygen species metabolic process. These terms were found to be enriched with genes such as *C5AR1, THBS1, SLC11A1, IL1B*, neurotrophic receptor tyrosine kinase 3 (*NTRK3*), prokineticin 2 (*PROK2*)*, FOS, PTGS2*, solute carrier family 8 member A1(*SLC8A1*) and aquaporin 9 (*AQP9*).

The most significantly enriched GO terms in the “cellular component” were the secretory granule membrane and the external side of plasma membrane. Within these terms, genes such as Fc gamma receptor IIa (*FCGR2A* also called *CD32)* and Fc gamma receptor IIIa (*FCGR3A* also called or *CD16*) were enriched. The significantly enriched GO terms were cytokine binding and immune receptor activity in the molecular function. The cytokine receptor genes *CXCR2* and interleukin 1 receptor type 2 (*IL1R2* also called *CD121b*), were enriched within these terms. **Supplementary Table S2** contains the details of the GO terms.

An analysis was conducted on the common DEGs list in the cornea and conjunctiva using the KEGG pathway enrichment method and the most significant KEGG pathways were identified (**Figure 2B**) (**Table 2**) (**Supplementary Table S3**). These were IL-17 signaling pathway, Hematopoietic cell lineage, Neutrophil extracellular trap formation, Phagosome, Complement and coagulation cascades, Cytokine-cytokine receptor interaction, Staphylococcus aureus infection, Pathogenic Escherichia coli infection, NF-kappa B signaling pathway, Toll-like receptor signaling pathway, TNF signaling pathway and Human cytomegalovirus infection.

**Table 2:** The KEGG pathways are ranked in order of likely biological relevance. q value = corrected p-value.

ID	Description	p-value	q-value	Gene ID
hsa04657	IL-17 signaling pathway	0.00035	0.0113	*FOS/CXCL8/PTGS2/IL1B*
hsa04640	Hematopoietic cell lineage	0.00042	0.0113	*CSF3R/IL1R2/ANPEP/IL1B*
hsa04613	Neutrophil extracellular trap formation	0.00055	0.0118	*FCGR2A/AQP9/C5AR1/NCF2/FCGR3A*
hsa04145	Phagosome	0.00211	0.0225	*FCGR2A/NCF2/THBS1/FCGR3A*
hsa04610	Complement and coagulation cascades	0.00366	0.0278	*C5AR1/ITGAX/PLAUR*
hsa04060	Cytokine-cytokine receptor interaction	0.00392	0.0278	*CSF3R/IL1R2/CXCR2/CXCL8/IL1B*
hsa05150	Staphylococcus aureus infection	0.00498	0.0288	*FCGR2A/C5AR1/FCGR3A*
hsa05130	Pathogenic Escherichia coli infection	0.00538	0.0288	*FCGR2A/FOS/CXCL8/IL1B*
hsa04064	NF-kappa B signaling pathway	0.00623	0.0288	*CXCL8/PTGS2/IL1B*
hsa04620	Toll-like receptor signaling pathway	0.00623	0.0288	*FOS/CXCL8/IL1B*
hsa04668	TNF signaling pathway	0.00803	0.0341	*FOS/PTGS2/IL1B*
hsa05163	Human cytomegalovirus infection	0.00856	0.0350	*CXCR2/CXCL8/PTGS2/IL1B*

q value = corrected p-value; ANPEP, alanyl aminopeptidase, membrane.

### 3.3 Significant differentially expressed genes in the cornea associated with corneal infection

A total of 512 differentially expressed genes were identified after comparing corneal infection samples and healthy volunteers’ samples based on the log fold change threshold criterion > 1 and adjusted P < 0.01. Of these, 508 genes were upregulated, and four were downregulated in the corneal infection samples (**Supplementary Table S4**).

The three most strongly upregulated genes in the cornea in response to infection were *CXCL8*, solute carrier family 2 member 3 (*SLC2A3*) and *TREM1*. While the downregulated genes were ribosomal protein S27a (*RPS27A*), ribosomal protein L13a (*RPL13A*), BAG cochaperone 1(*BAG1*) and pancreatic progenitor cell differentiation and proliferation factor (*PPDPF*).

The GO Enrichment analysis indicated that the significantly enriched “biological process” terms were relevant to neutrophil activation, complement receptor-mediated signaling pathway, extracellular structure organization, negative regulation of response to external stimulus and response to mechanical stimulus (**Figure 2C**). Examples of genes enriched within the Biological Process were *CXCL8, CXCR2, ITGAX, IL1B, TNF* receptor superfamily member 1B (*TNFRSF1B*), CD300a molecule (*CD300A*), formyl peptide receptor 1 and 2 (*FPR1*, *FPR2*), adhesion G protein-coupled receptor E3 (*ADGRE3*), alpha-2-macroglobulin (*A2M*), fibronectin leucine rich transmembrane protein 2 (*FLRT2*), ficolin 1 (*FCN1*), semaphorin 6A (*SEMA6A*), NLR family pyrin domain containing 3 (*NLRP3*), and the pattern recognition receptor (PRR) the toll-like receptor 4 (*TLR4*). Many genes that encode an extracellular matrix protein were upregulated and enriched within these terms, including tenascin C (*TNC*), laminin subunit alpha 1,2 and 3 (*LAMA1*, *LAMA2* and *LAMA3*), matrix metallopeptidase 10 and 25 (*MMP10* and *MMP25*). In addition, genes that related to the regulation of cell differentiation and proliferation were also upregulated, such as *FOS* and FosB proto-oncogene, AP-1 transcription factor subunit (*FOSB*).

The secretory granule membrane, cell-cell junction, and collagen-containing extracellular matrix were the most significantly enriched GO terms in the “cellular component”, with upregulated genes such as cell adhesion molecule 1 (*CADM1*), IL2 inducible T cell kinase (*ITK*), TIMP metallopeptidase inhibitor 3(*TIMP3*) and FRAS1-related extracellular matrix 1 (*FREM1*) enriched within these terms. Additionally, genes that contribute to epithelial cell-cell junctions and wound healing, such as desmoglein 3 (*DSG3*), tenascin C (*TNC*), collagen type XVII alpha 1 chain (*COL17A1*) and collagen type VI alpha 6 chain (*COL6A6*), were upregulated and enriched in the CC terms.

The GO terms significantly enriched in the molecular function category include metallopeptidase activity and cytokine binding. ADAM metallopeptidase domain 23 (*ADAM23*), chloride channel accessory 2 (*CLCA2*), *MMP10* and *MMP25*.Additionally, the genes are responsible for encoding the cytokines, and cytokines/chemokines receptors were enriched within the MF terms, such as interleukin 36 receptor antagonist (IL36RN), interleukin 5 receptor subunit alpha (*IL5RA*), *IL1R2* and *CXCR2*. **Supplementary Table S5** contains the details of the GO terms.

The KEGG pathway analysis revealed that the cornea’s differentially expressed genes were significantly enriched in only two pathways: the neuroactive ligand-receptor interaction pathway and the Staphylococcus aureus infection pathway (**Figure 2D**). Some examples of these genes include *C5AR1, FPR1*, and *FPR2*. The *Staphylococcus aureus* infection pathway showed more upregulated genes, including *FCGR2A*, keratin 14 (*KRT14*), and keratin 17 (*KRT17*).

### 3.4 Significant differentially expressed genes in the conjunctiva associated with corneal infection

In comparing conjunctiva samples from corneal infection patients with controls, 480 genes showed differential expression between the two groups. Among these genes, 477 were upregulated, and three were downregulated in the conjunctiva of patients with corneal infection (**Supplementary Table S6**). This determination was based on a threshold criterion of log fold change > 1.58 and adjusted P < 0.01.

The three most highly upregulated genes in the conjunctiva in response to infection were integrin subunit alpha X (*ITGAX*), myeloid cell nuclear differentiation antigen (*MNDA*) and pleckstrin (*PLEK*) triggering. While the three downregulated genes were ribosomal protein L6 (*RPL6*), HOP homeobox (*HOPX*) and ribosomal protein L32 (*RPL32*).

According to the GO Enrichment analysis, the top “biological process” terms that were significantly enriched relate to the inflammatory response, regulation of response to external stimulus, cytokine production, macrophage activation, and response to bacterium (**Figure 2E**). These immune response terms included genes like the pattern recognition receptor, the toll-like receptor 1(*TLR1*), and *TLR2*. In addition, chemokine genes and chemokine receptor genes were also upregulated: C-X-C motif chemokine ligand 1 (*CXCL1), CXCL2, CXCL8*, CXCR2 and C-C motif chemokine receptor 1(*CCR1*). Cytokine genes and cytokine receptors were also present in the immune terms, including *IL1B* and its receptor *IL1R2*, interleukin 1 receptor accessory protein (*IL1RAP*), interferon-gamma receptor 1(*IFNGR1*), interleukin 4 and 6 receptors (*IL4R* and *IL6R*), interleukin 1 receptor associated kinase 3 (*IRAK3*), additionally to the genes involved in the signaling cascades from these receptors, such as spleen-associated tyrosine kinase (*SYK*). The analysis also revealed the presence of cytokines production activator such as adhesion G protein-coupled receptor E2 (*ADGRE2*) and the cytokine signaling negative regulators, the suppressor of cytokine signaling 3 (*SOCS3*), all of which were enriched within the immune response terms. Genes associated with cell migration and genes promoting cell adhesion, such as FGR proto-oncogene, Src family tyrosine kinase (*FGR*) and the integrins *ITGAM* and *ITGAX*, were also enriched. The genes that related to the regulation of inflammatory response were also involved in the immune response terms like phosphoinositide-3-kinase adaptor protein 1 (*PIK3AP1*), MEFV innate immunity regulator, pyrin (*MEFV*), TNF receptor superfamily member 1A (*TNFRSF1A*) and TNF superfamily member 14 (*TNFSF14*).

The angiogenesis and wound healing terms were also identified as biological process enriched terms. Those genes that showed considerable differences in expression levels within this pathway included notch receptor 1 (*NOTCH1*) and integrin subunit alpha 5 (*ITGA5*). Regarding cellular components, the secretory granule membrane and intrinsic component of plasma membrane were the most significantly enriched GO terms. CD53 molecule (*CD53*), CD93 molecule (*CD93*), CD46 molecule (*CD46*), CD59 molecule (*CD59*), CD163 molecule (*CD163*) and transforming growth factor beta receptor 1 (*TGFBR1*) were found to be upregulated and enriched in the CC terms. Further analysis of differentially expressed genes determined that their primary molecular function is related to signaling receptor activity and immune receptor activity. **Supplementary Table S7** contains the details of the GO terms.

An analysis was carried out on the DEGs list in the conjunctiva using the KEGG pathway enrichment method. As a result, the most significant KEGG pathways were identified, including Leishmaniasis, Chemokine signaling pathway, Complement and coagulation cascades, NF-kappa B signaling pathway, TNF signaling pathway, RIG-I-like receptor signaling pathway, Viral protein interaction with cytokine and cytokine receptor, Th17 cell differentiation, Th1 and Th2 cell differentiation, Growth hormone synthesis, secretion and action, NOD-like receptor signaling pathway and JAK-STAT signaling pathway (**Figure 2F**). **Supplementary Table S8** contains the details of the KEGG pathway identified genes.

### 3.5 Tear cytokine analysis

Out of total 38 corneal infection tear samples analyzed, microbial diagnostic results could be identified for 33, 12 (36%) of which were culture positive. Seven were gram-positive bacteria and five gram-negative bacteria with six cases of polymicrobial (6/12, 50%) keratitis, usually with Herpes Simplex or Varicella Zoster Virus (**Supplementary Table S9**).

**Figure 3:**
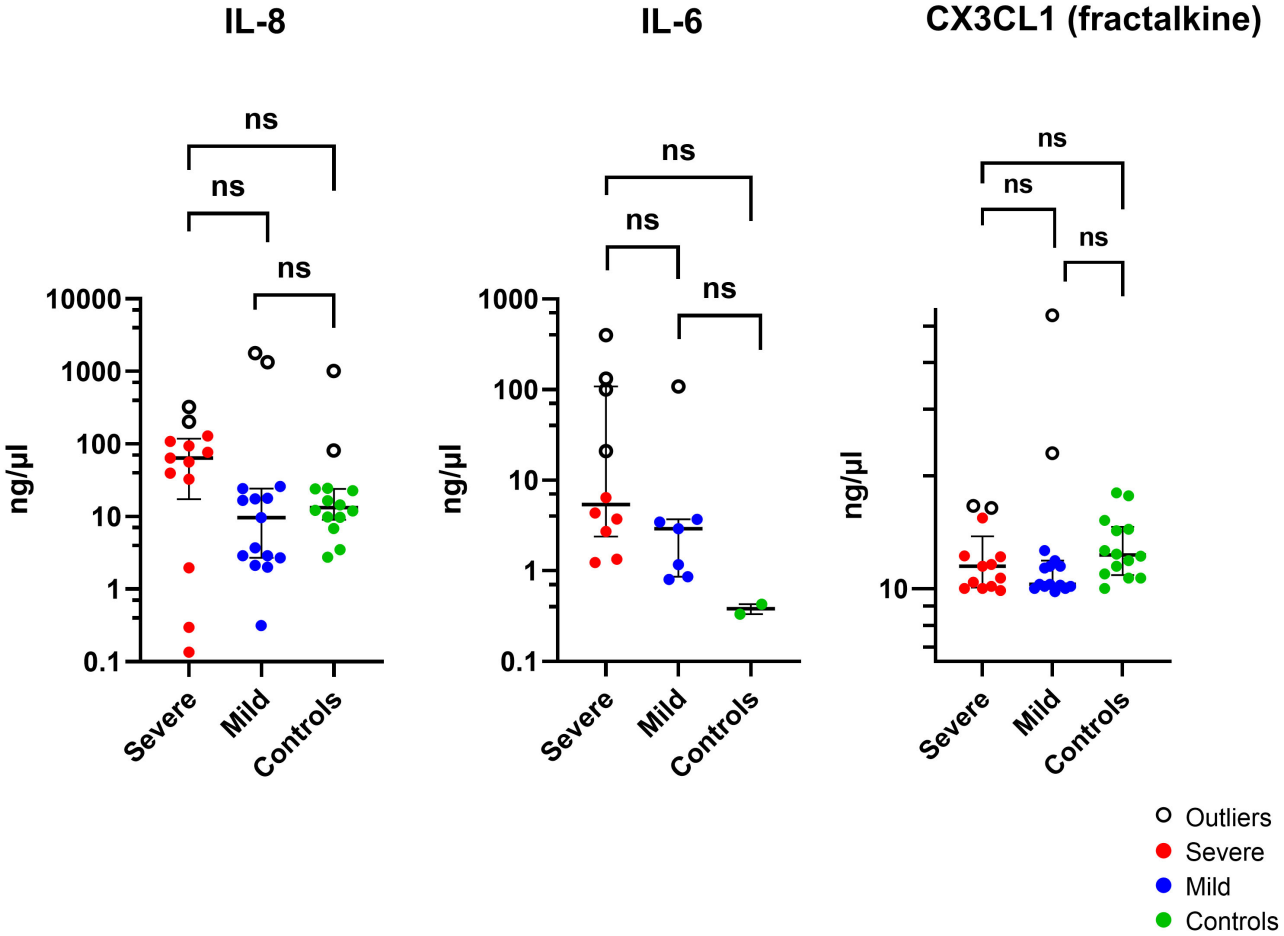
The Log scale scatter plot displays the median ± IQR of tear cytokine levels among individuals with severe and mild corneal infections and controls. Statistical significance between unpaired samples was assessed using a two-tailed Mann-Whitney non-parametric test. NS, not significant (corrected P >0.05).

The cytokine concentrations (median and interquartile ranges) were determined for the case and control tear samples, including mild and severe cases versus controls, and severe versus mild cases. Severe keratitis was defined as the final best-corrected visual acuity (BCVA) of ≤ 20/40, repeat scrape or culture positive. Cases of microbial keratitis that did not meet these criteria were classified as mild. Medians and interquartile ranges were tabulated due to the skewed data distribution **Table 3**. The concentration of IL-8 was slightly but not significantly elevated in corneal infection samples compared to the control group (P > 0.05). The concentration of IL-6 was not significantly higher in corneal infection samples than in the controls after applying the Bonferroni correction for multiple comparisons (P = 0.0067) (corrected P = 0.0536). Patients with severe corneal infection (n = 10) exhibited slightly but significantly higher tear concentrations of IL-6 compared to the controls (P = 0.0303) (corrected P = 0.2424) (**Figure 3**). As previously mentioned, severe keratitis is defined as having a repeat scrape or positive culture and a final best-corrected visual acuity (BCVA) of ≤ 20/40. There were also no statistically significant differences in the concentration of IL-6 in tears of patients with mild corneal infection (n = 7) compared to the controls and between the severe and mild groups (P = 0.0556) (corrected P = 0.4448) and (P = 0.0878) (corrected P = 0.7024), respectively. IL-1β levels were observed in the patients’ samples; however, they were below the minimum detectable limit in the control samples. Therefore, it was concluded that performing the Mann-Whitney test was not feasible. The study results indicated that CX3CL1 was detected in all participants with no significant variations between the groups. Conversely, in a small number of cases the cytokines IL-10, IL-12 (p70), IL-17A, and IL-23 could be detected, but these cytokines were below the minimum limit of detection in most of the case and control samples. Therefore, it was not feasible to perform the Mann-Whitney test, except for IL-23, which was detected in two control samples (P > 0.9999). The proportion of detectable samples for each cytokine is outlined in **Table 4**.

**Table 3:** Cytokine expression in tears of corneal infection patients and healthy volunteers.

Tear Cytokine	Corneal Infection Patients (Median ng/µl & IQR)	Severe (Median ng/µl & IQR)	Mild (Median ng/µl & IQR)	Controls (Median ng/µl & IQR)
IL-8	25.0 (3.0; 98.0)	64.0 (17.0; 119.0)	10.0 (3.0; 24.0)	13.0 (9.0; 24.0)
IL-6	4.0 (1.0; 21.0)	5.0 (2.0; 108.0)	3.0 (0.9; 4.0)	0.4 (0.3; 0.4)
IL-1β	0.3 (0.2; 1.0)	0.3 (0.1; 0.8)	0.3 (0.2; 700.0)	0
CX3CL1 (fractalkine)	11.0 (10.0; 11.0)	11.0 (10.0; 12.0)	10.0 (10.0; 11.0)	11.0 (10.0; 12.0)
IL-10	0.9 (0.8; 1.0)	0	0	0
IL-12 (p70)	0.2 (0.1; 0.2)	0	0	0
IL-17A	0.4 (0.1; 0.4)	0	0	0
IL-23	17.0 (4.0; 48.0)	0	0	21.0 (4.0; 37.0)

IQR, interquartile ranges.

**Table 4:** The proportion of detectable Samples for corneal infection patients and healthy volunteers.

Tear Cytokine	Corneal Infection Patients, n (%)	Severe Cases, n (%)	Mild Cases, n (%)	Controls, n (%)
IL-8	38/38 (100)	13/13 (100)	15/15 (100)	14/14 (100)
IL-6	23/38 (60.5)	10/13 (76.9)	7/15 (46.7)	2/14 (14.3)
IL-1β	16/38 (42.1)	7/13 (53.8)	5/15 (33.3)	0
CX3CL1 (fractalkine)	38/38 (100)	13/13 (100)	15/15 (100)	14/14 (100)
IL-10	2/38 (5.3)	0	0	1/14 (7.14)
IL-12 (p70)	4/38 (10.5)	0	0	0
IL-17A	3/38 (7.9)	0	0	0
IL-23	4/38 (10.5)	0	0	2/14 (14.3)

The RNA cohort had higher levels of CX3CL1 (P = 0.02; corrected P = 0.16) and IL-1β (P = 0.02 corrected P = 0.16) compared to the tear film only cohort, but there was no difference in IL-6 (P = 0.08) and IL-8 (P = 0.17) levels, performed with Mann-Whitney test.

After excluding outliers shown in (**Figure 3**) (**Supplementary Table S10**) using the ROUT method, the analysis revealed that there was no statistically significant variation in the concentration of IL-8 protein in tears of patients with corneal infection as compared to the control group (P = 0.7184). A higher concentration of IL-6 was observed in the samples of corneal infection patients compared to the control samples (P = 0.0117; corrected P = 0.0936), but the difference was not statistically significant (P > 0.05). There was also no significant difference observed between the severe and mild groups and the control group (P = 0.0714). Moreover, the concentration of CX3CL1 was higher in corneal infection patients’ samples than in the control group (P = 0.0017; corrected P = 0.0136). Individuals with mild corneal infection had significantly higher CX3CL1 concentrations than the control group (P = 0.0049; corrected P = 0.0392). Nevertheless, there wasn’t any statistically significant difference in the concentration of CX3CL1 in the tears of patients with severe corneal infection compared to healthy volunteers or between the severe and mild groups (P = 0.0505) and (P = 0.5201), respectively.

## 4 Discussion

The study has identified genes, biological processes and pathways that may play a pivotal role in the pathogenesis of corneal infection. Differential expression of genes was observed in both the cornea and conjunctiva of corneal infection patients compared to controls. The upregulated genes that overlapped in both the cornea and conjunctiva had primary associations to immune response, regulation of angiogenesis, and apoptotic signaling pathways. These pathways align with the known biological pathways of keratitis progression (Zhou et al., 2010; Narimatsu et al., 2019; Shrestha et al., 2021; Lin et al., 2022).

*CXCL8* (encodes the IL-8 protein) had the most significant upregulation among the differentially expressed genes (DEGs) in the cornea of patients with corneal infection and was upregulated in the conjunctiva. An increased concentration of the IL-8 protein was found in the tears of patients compared to controls, but the difference observed was not statistically significant. *CXCL8* is expressed by various ocular surface cell types, including corneal and conjunctival epithelial cells, corneal keratocytes, PMNs, and macrophages, in response to the infection (Elner et al., 1991; Oakes et al., 1993; Scapini et al., 2000; Venza et al., 2007; Waugh and Wilson, 2008; Shtein et al., 2012). Animal studies have shown that homologues of IL-8, such as macrophage inflammatory protein-2 (also known as CXCL2) and keratinocyte-derived chemokine (also known as CXCL1), are also highly expressed in the corneas of rats and mice infected with *Pseudomonas aeruginosa* or *Staphylococcus aureus* (Cole et al., 2000; Xue et al., 2003; Hume et al., 2005). As well, the receptor for these CXC chemokines, CXCR2, which was significantly upregulated in the cornea and conjunctiva of the corneal infection patients, plays a significant role in the pathogenesis of bacterial keratitis (Khan et al., 2007; Cole et al., 2014). These studies in animals have shown that CXC chemokines are produced by corneal epithelial cells and infiltrating neutrophils (Cole et al., 2000; Cole et al., 2005) (the conjunctiva of animals was not studied). Furthermore, studies blocking the action of CXCL2 demonstrated its involvement in the neovascularisation of the cornea and recruitment of neutrophils (Xue et al., 2002; Xue et al., 2003).

Cell culture of human corneal epithelial cells confirmed that *CXCL8* was also able to be upregulated when the cells were exposed to *P. aeruginosa* (Xue et al., 2000). Human corneal stromal cells exposed to lipopolysaccharide (LPS) display a time-dependent expression of IL-8 (Shtein et al., 2012). An *in vitro* study on human adenovirus keratitis found that the IL-8 produced in the process binds to the basement membrane of epithelial tissues, creating tissue reservoirs (Rajaiya et al., 2015).

Polymorphonuclear neutrophils (PMNs) attracted to persistent reservoirs produce MMPs, which result in collagen degradation (Ghasemi et al., 2011). IL-8 plays a crucial role in optimizing this process by increasing MMP production and activation in PMNs (Li et al., 2003a; Ellis et al., 2018; Raziyeva et al., 2021). A previous study has shown that IL-8 plays a role in upregulating the mRNA expression of MMP-2 and MMP-9 in human endothelial cells (Li et al., 2003b).This might have been involved in the upregulation of *MMP10* and *MMP25* in the cornea and *MMP25* in the conjunctiva in the current study.

The ocular surface, particularly the cornea, is known to have a certain level of immune privilege (de Paiva et al., 2022) due to the absence of blood vessels. On the other hand, the conjunctiva possesses immunity and competence (de Paiva et al., 2022). The conjunctiva and to a lesser extent, the cornea are responsible for determining whether to initiate protective mechanisms or tolerate insults (de Paiva et al., 2022). Critical components of conjunctival mucosal tolerance in the ocular surface include CD11c+ antigen-presenting cells (APCs) (Dang et al., 2014; de Paiva et al., 2022). In this study, among the differentially expressed genes in the conjunctiva, *ITGAX* (*CD11c*) was the most upregulated. The upregulation of this gene was also observed in the cornea but to a lesser degree. Significant upregulation of *ITGAX* in patients with late-stage microbial keratitis has been reported compared to normal corneal tissue (Chidambaram et al., 2017). These elevated expression levels may be attributed to the expression of *CD11c* on myeloid dendritic cells or macrophages (Merad et al., 2013; Mrugacz et al., 2021). This gene was a member in many GO terms in the current study, including inflammatory response, neutrophil activation and degranulation, extracellular structure organization, signaling receptor activity, and angiogenesis. A study has revealed that *CD11c* expression is notably elevated in individuals with atopic keratoconjunctivitis, a condition characterized by persistent inflammation of the conjunctiva (Matsuda et al., 2019). The macrophage response and phenotype were analyzed in a murine model of corneal inflammation. It was observed that macrophages accumulated on both the corneal endothelium and stroma (Chinnery et al., 2014). According to a study on mice, the central and peripheral corneas showed a higher density of dendritic cells during the initial stages of Herpes simplex virus type 1 keratitis (Jamali et al., 2020).

*CD11c* has been identified as a significant contributor to the cornea’s immune response and inflammatory processes during *P. aeruginosa* infection. Previous murine studies have demonstrated the crucial role of *CD11c* in preventing adhesion and penetration of *P. aeruginosa* in healthy cornea (Metruccio et al., 2017). *CD11c* has also been shown to play a significant role in activating immune and inflammatory responses during *P. aeruginosa* infections, thus contributing to the defense against the pathogen (Metruccio et al., 2017). Recent research on murine subjects has demonstrated that *CD11c* plays a significant role in regulating the neutrophil maturation process and its associated effector functions (Hou et al., 2023). In a mouse study on *P. aeruginosa* corneal infections, initiating apoptosis of neutrophils at an earlier stage resulted in better disease outcomes (Zhou et al., 2008). Considering this, targeting the *CD11c* to control the neutrophil maturation process during corneal infections could be a promising strategy for enhancing disease outcomes. While the present study has identified significant transcriptional changes in *CD11c* in the conjunctiva and cornea of corneal infection patients, it is necessary to conduct further investigation to determine whether these gene expression changes correspond to protein expression differences.

Evidence suggests that besides *CD11c, TREM1* is also a critical receptor in regulating immune responses during the early stages of bacterial infection (Bleharski et al., 2003; Colonna, 2003; Lagler et al., 2009; Hommes et al., 2014; Colonna, 2023). The current study has unveiled a significant upregulation in *TREM1* in both the cornea and conjunctiva of the corneal infection patient. A study on the *P. aeruginosa* murine keratitis model showed that *TREM1* has an inflammatory amplification effect during infection (Wu et al., 2011). *TREM1* expression is detectable in different types of immune cells, such as PMNs, monocytes, and macrophages (Bouchon et al., 2000; Schenk et al., 2007; Ferat-Osorio et al., 2008), which is likely the driver of the increased expression in the present study. *TREM1* stimulates the release of IL-8 and induces the upregulation of adhesion molecules, including *CD11c* (Bouchon et al., 2000). These findings suggest that *TREM1* may be involved in the upregulation of these genes in the current study. In cases of unilateral bacterial keratitis, there is a notable elevation in the concentration of *TREM1* in both eyes (Yamaguchi et al., 2014). Blocking *TREM1* in a mouse model of *P. aeruginosa* keratitis resulted in a reduction of corneal disease severity (Wu et al., 2011). Conversely, activating *TREM1* signaling exacerbated the disease, leading to an earlier corneal perforation (Wu et al., 2011). Further research is required to assess the viability of T*REM1* as a diagnostic indicator for determining the extent of corneal infections and predicting unfavorable outcomes.

The findings of the current study indicate that most downregulated genes in both the cornea and conjunctiva were those encoding ribosomal proteins. It is noteworthy that among the identified genes, *RPS27A* plays a crucial role in the biogenesis of ribosomes and cellular growth (Nosrati et al., 2015; Zhou et al., 2015). *RPS27A* has been shown to exert a regulatory effect on cell cycle progression, promote proliferation, and have an inhibition effect on apoptosis of leukemia cells (Wang et al., 2014). In addition to ribosomal protein genes, this study found that the *BAG1* was downregulated in the cornea. *BAG1* is known to possess anti-apoptotic properties and may have a significant role in preventing cell death through autophagy activation (Portt et al., 2011), which is the cellular mechanism of recycling (Kamil and Mohan, 2021). The reduced expression of ribosomal protein genes and *BAG1* in the cornea suggests that corneal cells could potentially be at a higher vulnerability to apoptotic triggers (Portt et al., 2011). In the current study, *PPDPF* is identified as one of the down-regulated genes in the cornea. Previous research has shown that *PPDPF* is expressed in the corneal limbus of healthy individuals (Li et al., 2022). This may indicate the important role of this gene in regulating corneal cell differentiation and proliferation. In a previous study conducted on rats, it was found that the expression level of this gene increased with the onset of cataracts in an ex vivo model (Nagaya et al., 2022). Cataracts are an eye condition that causes cloudiness and opaqueness of the lens (Nagaya et al., 2022). Whereas, in conjunctiva, the study found that the expression of *HOPX* was significantly reduced, in addition to the ribosomal protein genes. As part of the investigation into the differentiation of limbal stem cells in homeostasis, a significant increase in the expression of the *HOPX* was observed in wing cells (Lin et al., 2023). This indicates that *HOPX* plays a crucial role in regulating the differentiation and regenerative processes of epithelial tissues, as seen in other similar tissues (Yang et al., 2010; Ota et al., 2018). Silencing HOPX in human keratinocytes (Yang et al., 2010) and murine alveolar epithelial cell lines (Ota et al., 2018) increases cellular proliferation (Ota et al., 2018) and differentiation markers (Yang et al., 2010). Taken together, these findings suggest that corneal infection results in an increase in apoptosis and a decrease in differentiation compared to a non-infected cornea. The apoptosis of the keratocytes can serve as a protective mechanism against the posterior spread of infectious pathogens in corneal injuries (Wilson and Esposito, 2009). These apoptosis signals attract inflammatory cells to the area, which are crucial for healing (Yoon et al., 2020; Kamil and Mohan, 2021). Further research is required to establish the exact role of *BAG1, PPDPF*, *HOPX*, and ribosomal protein genes in the cornea and conjunctiva, both in normal and pathological states.

A further objective of this current research was to analyze cytokines and chemokines levels in the tears of patients with corneal infections. The study compared those with mild infection to those with severe infection and healthy controls. The results revealed that although the difference in IL-8 concentration did not show statistical significance, it is noteworthy that the levels were relatively higher in patients than in the control group. This finding was consistent with RNA-seq results. However, mRNA-protein correlation can depend upon factors such as post-transcriptional regulation, including protein degradation and protein buffering which may contribute to this imbalance (Buccitelli and Selbach, 2020). Furthermore, IL-6 was significantly detected in the tears of patients with severe corneal infections compared to healthy volunteers, but it was not statistically significant after the Bonferroni correction. Previous research has shown that increased levels of IL-8 and IL-6 have been observed in patients suffering from bacterial keratitis caused by gram-negative or gram-positive bacteria compared to healthy controls (Yu et al., 2012; Wu et al., 2021). Moreover, the levels of IL-8 and IL-6 reported in these studies were higher than those observed in the present study. The presence of CX3CL1 was observed in all study participants. However, no statistically significant variances were observed between the groups and did not appear in this study’s list of genes that were differentially expressed. However, IL-10, IL-12 (p70), IL-17A, and IL-23 were detected in only a small number of cases and were found to be below the minimum limit of detection in most samples. These cytokines were not found in the list of genes that exhibited differential expressions. The findings of the present study align with previous research on bacterial keratitis, indicating that IL-17A levels were not significantly increased in the affected eye compared to healthy controls (Powell et al., 2010). However, the study found that IL-17A levels were significantly elevated in the unaffected contralateral eyes (Powell et al., 2010). These results suggest that IL-17A may play a role in the pathogenesis of bacterial keratitis, and further research is required to understand its effects fully. The level of IL-10 and IL-12 (p70) in the tear of bacterial keratitis patients was higher in previous studies (Yu et al., 2012), than in this study. Differences observed between the current study’s cytokines levels and previous research might be due to the sample size. Therefore, a more comprehensive investigation of these proteins with a larger sample size was required.

Although there are few studies on human microbial keratitis gene expression, this study’s results show some similarity to previous ocular expression studies. Earlier studies on expressed genes in human corneal tissue and scrapings showed that bacterial keratitis leads to an increase in the expression of *TLR4* and *TLR2* genes (Karthikeyan et al., 2013; Chidambaram et al., 2017; Tian et al., 2020). The findings of this study are consistent with previous research, revealing significant regulation of *TLR4*. In the conjunctiva, *TLR1* and *TLR2* are also regulated. The current study identified 47 upregulated genes overlapping between the cornea and conjunctiva. Notably, 21 of these genes were previously listed in the transcriptional profile of the late-stage human bacterial keratitis microarray study (Chidambaram et al., 2017). Interestingly, these genes included *CXCL8, ITGAX, TREM1, IL1B*, *SLC11A1, C5AR1, PTGS2, G0S2, AQP9, IL1R2*, and *CD163*. It is worth noting that another 21 genes were also upregulated, specifically in the cornea in the current study and the previous study (Chidambaram et al., 2017). These genes included *NLRP3, FOSB, FPR1, FPR2*, and *MMP10*. There were notable dissimilarities in the findings of both studies. It’s worth noting that while the previous study (Chidambaram et al., 2017) found that among the chemokines, *CXCL2* showed the highest fold change, the current study observed up-regulation of genes for *CXCR2, THBS1, FOS* and *MMP25*, which were not included in the gene list of the previous study (Chidambaram et al., 2017). *THBS1* and *FOS* play essential roles in the process of tissue regeneration and wound healing (Verrier et al., 1986; Blanco-Mezquita et al., 2013). The precise role of *MMP25* in wound healing is not yet fully understood (Caley et al., 2015). Nonetheless, its expression in leukocytes suggests that *MMP25* may significantly influence IL-8 secretion and respiratory bursts (Cui et al., 2017). The observed distinction may be attributable to the use of microbial keratitis corneal tissues for the microarray study, whereas the control incorporated normal cadaver corneas. Furthermore, the limitations of microarray technology, such as cross-hybridization issues and probe selection biases, could also contribute to the disparity (Liu et al., 2007; Roy et al., 2011). RNA sequencing can provide an unbiased identification of all transcripts in a sample, offering a more comprehensive gene expression analysis (Liu et al., 2007; Roy et al., 2011). Moreover, the observed differences between the two studies could also be due to a heightened emphasis on late-stage keratitis within a median of 15 days following the onset of symptoms in the previous study. These differences highlight the need for continued research to understand the complex mechanisms of microbial keratitis pathogenesis.

A recent study analyzed the molecular changes in the human cornea during bacterial/fungal keratitis using RNA sequences (Lapp et al., 2024). It is worth noting that 31/47 genes upregulated in the cornea and conjunctiva in the current study were also found to be significantly upregulated in this recent study using similar RNA sequence technology (Lapp et al., 2024). These genes included *CXCL8, ITGAX, TREM1, CD163, C5AR1, CXCR2, MMP25* and *THBS1.* Two of the 47 upregulated genes in the current study were significantly downregulated in the recent RNA sequences study (Lapp et al., 2024). However, the function of these genes is unlikely to be important in this context. These differences in differentially expressed genes are likely affected by the limited sample size in both studies, differences in the sample processing techniques and the nature of the samples. For example, the current study collected swab samples from the corneal lesion of the patients at the time of diagnosis. Meanwhile, in the previous research, corneal tissue was obtained from people who had undergone keratoplasty, where the tissue was processed by fixation in formalin and subsequent embedding in paraffin (Lapp et al., 2024). Even with these differences, the upregulated genes of both studies show significant similarities. This suggests a common immune response mechanism to microbial keratitis and provides a strong basis for further research into the mechanisms of microbial keratitis pathogenesis.

There are several limitations of the current study. Due to the limited samples available, the statistical power of the experiment was restricted. To ensure a more comprehensive analysis, additional samples from both corneal infection patients and controls are required. It is unknown specifically what type of cells are collected with the swabs, nor the exact number of cells. However, the major cell type that was most probably collected is superficial epithelial cells (Anagnostopoulou-Fotinopoulou and Rammou-Kinia, 1993; Kobayashi et al., 1997; Yağmur et al., 1997). Furthermore, only two of seven corneal infection samples were culture-positive for bacteria. Several research studies have conducted comparative analyses of keratitis cases, including culture-positive and culture-negative cases (Sharma et al., 2007; Bhadange et al., 2015; Atta et al., 2023). According to these studies, several possible reasons exist for the absence of microbial growth in culture-negative cases. These reasons include using antimicrobials before presentation, inadequate specimen collection due to the smaller size of the defects, and the unavailability of appropriate types of culture (Sharma et al., 2007; Bhadange et al., 2015; Atta et al., 2023). The positivity rate of 40% in the current is within normal limits of corneal cultures (Ng et al., 2015; Oliveira-Ferreira et al., 2019). While positive corneal culture may have different presenting and prognostic factors, a larger sample size would be required to determine differences between culture positive and negative cases gene expression. A strength of the current study was the ability to detect gene expression and to part replicate the results in protein concentration for some of the targets. To better comprehend the mechanisms underlying the observed changes in gene expression, it is crucial to conduct targeted gene expression analysis of immune response to culture positive and culture negative, gram-positive and gram-negative bacteria, as well as patient severity outcomes.

## 5 Conclusion

This study investigated the changes in the immune transcriptomic profile in the cornea and conjunctiva, as well as the tear cytokines of patients with corneal infection. The study findings revealed a significant overlap in the differential gene expression observed in the cornea and conjunctiva and identified a set of genes and pathways that play a role in corneal infection pathogenesis. It was also observed that the corneal infection genes were highly associated with immune response, regulation of angiogenesis, and regulation of apoptotic signaling pathways. Although this study observed moderate alignment between gene and tear protein expression, the integration of multi-omics datasets in future studies is likely to provide a better understanding of the complex genome to phenotype relationship and hold insights for better therapeutics. This study’s outcomes can offer valuable insights for further research. In future, these may contribute to the development of targeted therapeutic interventions to reduce tissue damage and control infection severity by targeting the underlying genetic and immunological factors associated with corneal infection.

## Data availability statement

The datasets presented in this study can be found in online repositories. The names of the repository/repositories and accession number(s) can be found below: https://www.ncbi.nlm.nih.gov/bioproject/PRJNA1050754. Repository, accession number PRJNA1050754.

## Ethics statement

The studies involving humans were approved by the Western Sydney Local Health District Human Research Ethics Committee (LNR/16/WMEAD/401), and the research followed the tenets of the Declaration of Helsinki. All participants signed informed consent prior to study commencement. The studies were conducted in accordance with the local legislation and institutional requirements. The participants provided their written informed consent to participate in this study. Written informed consent was obtained from the individual(s) for the publication of any potentially identifiable images or data included in this article.

## Author contributions

HA: Data curation, Formal Analysis, Investigation, Software, Writing – original draft, Writing – review & editing. GP: Methodology, Supervision, Writing – review & editing. SS: Methodology, Writing – review & editing. JO: Supervision, Writing – review & editing. MW: Supervision, Writing – review & editing. AW: Supervision, Writing – review & editing. NC: Conceptualization, Funding acquisition, Methodology, Project administration, Resources, Supervision, Writing – review & editing.
