# Supplementary material for: Ocular surface immune transcriptome and tear cytokines in corneal infection patients
Source: Front Cell Infect Microbiol. 2024 Apr 17;14:1346821. doi: 10.3389/fcimb.2024.1346821 (PMC11061372; doi:10.3389/fcimb.2024.1346821)
Supplement: Supplementary file 1 [file DataSheet_1.docx]

Supplementary Material

Ocular Surface Immune Transcriptome and Tear Cytokines in Corneal Infection Patients

Heba Alenezi 1,2,4*, Grant Parnell 3, Stephen Schibeci 3, Jerome Ozkan 2, Mark Willcox 2, Andrew White 4, Nicole Carnt 2,4,5

^1 Department of Medical Laboratory Sciences, College of Applied Medical Sciences in Al-Kharj, Prince Sattam Bin Abdulaziz University, Al-Kharj 11942, Saudi Arabia.^

^2 School of Optometry and Vision Science, The University of New South Wales, Sydney 2033, NSW, Australia^

^3 Centre for Immunology and Allergy Research, Westmead Institute for Medical Research, The University of Sydney, Sydney 2145, NSW, Australia^

^4 Centre for Vision Research, Westmead Institute for Medical Research, The University of Sydney, Sydney 2145, NSW, Australia^

^5 Institute of Ophthalmology, University College London, London EC1V 9EL, United Kingdom^

*** Correspondence:** Heba Alenezi: hh.alenezi@psau.edu.sa

## Supplementary Figure

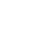

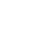


**Supplementary Figure 1.** Principal Component Analysis (PCA) from the RNA-seq data of the samples. Corneal infection samples are in blue, and samples from healthy volunteers are in red. Corneal samples are in a triangle shape, and Conjunctiva samples are in a circle shape.
